# The default mode network is disrupted in parkinson's disease with visual hallucinations

**DOI:** 10.1002/hbm.22577

**Published:** 2014-07-01

**Authors:** Nailin Yao, Richard Shek‐Kwan Chang, Charlton Cheung, Shirley Pang, Kui Kai Lau, John Suckling, James B. Rowe, Kevin Yu, Henry Ka‐Fung Mak, Siew‐Eng Chua, Shu Leong. Ho, Grainne M. McAlonan

**Affiliations:** ^1^ Department of Psychiatry Queen Mary Hospital, The University of Hong Kong Pokfulam Hong Kong; ^2^ Division of Neurology, Department of Medicine The University of Hong Kong, Queen Mary Hospital Pokfulam Hong Kong; ^3^ State Key Laboratory for Cognitive Sciences The University of Hong Kong Pokfulam Hong Kong; ^4^ Department of Psychiatry and Behavioural and Clinical Neuroscience Institute University of Cambridge, and Cambridge and Peterborough Foundation NHS Trust Cambridge United Kingdom; ^5^ Department of Clinical Neurosciences and Behavioural and Clinical Neuroscience Institute University of Cambridge United Kingdom and Medical Research Council Cognition and Brain Sciences Unit Cambridge, United Kingdom; ^6^ Department of Diagnostic Radiology The University of Hong Kong Pokfulam Hong Kong; ^7^ Department of Forensic and Neurodevelopmental Science Institute of Psychiatry, King's College London London SE5 8AZ United Kingdom

**Keywords:** resting, function, MRI, cortical thickness, coactivation

## Abstract

Background: Visual hallucinations (VH) are one of the most striking nonmotor symptoms in Parkinson's disease (PD), and predict dementia and mortality. Aberrant default mode network (DMN) is associated with other psychoses. Here, we tested the hypothesis that DMN dysfunction contributes to VH in PD. Methods: Resting state functional data was acquired from individuals with PD with VH (PDVH) and without VH (PDnonVH), matched for levodopa drug equivalent dose, and a healthy control group (HC). Independent component analysis was used to investigate group differences in functional connectivity within the DMN. In addition, we investigated whether the functional changes associated with hallucinations were accompanied by differences in cortical thickness. Results: There were no group differences in cortical thickness but functional coactivation within components of the DMN was significantly lower in both PDVH and PDnonVH groups compared to HC. Functional coactivation within the DMN was found to be greater in PDVH group relative to PDnonVH group. Conclusion: Our study demonstrates, for the first time that, within a functionally abnormal DMN in PD, relatively higher “connectivity” is associated with VH. We postulate that aberrant connectivity in a large scale network affects sensory information processing and perception, and contributes to “positive” symptom generation in PD. *Hum Brain Mapp 35:5658–5666, 2014*. © **2014 The Authors Human Brain Mapping Published by Wiley Periodicals, Inc.**

## INTRODUCTION

Parkinson's disease (PD) is often regarded as a motor disorder, but nonmotor manifestations also occur, and indeed are more distressing for many individuals [Gibson et al., 2013]. Of these, one of the most common and striking features is visual hallucinations (VH) [Bernal‐Pacheco et al., 2012; Rabey, 2009]. VH affect 20–75% of patients with PD [Diederich et al., 2009], and predict dementia and mortality [Aarsland et al., 2007; Fenelon et al., 2000]. Clinically, VH in PD often involve transient complex figures of people, animals, or objects during mind wandering [Diederich et al., 2009]. However, the neuropathological mechanism remains unclear.

In other psychotic conditions such as schizophrenia, hallucinations are linked with dysfunction of the default mode network (DMN) [Jardri et al., 2013; Lutterveld et al., 2014]. The medial prefrontal cortex and precuneus/posterior cingulate gyrus serve as hubs within the DMN, while adjacent regions such as the medial temporal lobe and inferior parietal lobe are also recruited [Di and Biswal, 2014; Raichle et al., 2001]. The DMN is a “task‐negative” network which shows greater activity when individuals are at rest [Raichle et al., 2001], and are involved in endogenous cognitive activity such as self‐referential thoughts and (visual) memory retrieval [Buckner et al., 2008; Olypher et al., 2006].

In PD and other psychiatric disorders, VH are thought to result from unconstrained “top‐down” processing of endogenous or “intrinsic” memories and attention [Bar et al., 2006; Behrendt, 2010; Onofrj et al., 2013]. Given that the DMN is essential for intrinsic memory [Buckner et al., 2008], we postulated that VH in PD are at least partially explained by the disruption of the DMN leading to aberrant intrinsic memory processes [Diederich et al., 2009]. Consistent with this, there is preliminary evidence that pathology in PDVH includes Lewy body accumulation in the regions of DMN responsible for memory [Gallagher et al., 2011; Ramirez‐Ruiz et al., 2008]; and a recent study showed that PD patients without cognitive impairment have functional disruption in the DMN, [Tessitore et al., 2012]. Confirmation of the relationship between the DMN and VH in PD in PDVH is, therefore, potentially illuminating [Tessitore et al., 2012], but has not been directly examined.

For the first time, to our knowledge, an independent component analysis (ICA) of patterns of low‐frequency brain activity was carried out to examine the DMN in PDVH [Beckmann et al., 2005; Uddin et al., 2013]. ICA is a data‐driven, unbiased approach for uncovering coherent and highly reproducible large‐scale brain networks [Damoiseaux et al., 2006], and is considered a useful option to compare large‐scale brain networks between groups [Filippini et al., 2009; Zuo et al., 2010]. The groups were matched for age, gender, and general cognitive ability [measured using mini‐mental state examination (MMSE)]. We postulated that DMN pathology would be prominent in PDVH compared to both PDnonVH and healthy control group (HC). In a supplemental analysis, we also looked at regional cortical thickness to determine whether predicted DMN dysfunction would be accompanied by structural change.

## MATERIALS AND METHODS

### Participants

PD was diagnosed according to the criteria of the UK Parkinson's Disease Society Brain Bank. The assessments included: Hoehn and Yahr Scale [Hoehn and Yahr, 1967] to assess stage of illness; Unified Parkinson's Disease Rating Scale [UPDRS; Fahn et al., 1987], part III to evaluate motor symptom severity; Montgomery–Åsberg Depression Rating Scale—Self‐assessment (MADRS‐S) [Svanborg and Asberg, 1994] to rate depressive symptoms; MMSE [Folstein et al., 1975] to assess cognitive impairment; the positive and negative syndrome scale [Kay et al., 1987] and the Parkinson psychosis rating scale (PPRS) [Friedberg et al., 1998] to assess psychosis. The PPRS includes a detailed description of VH recorded from patients and caregivers. Participants were matched for age (>55 years), gender, and MMSE score. One person with PD experienced transient VH several months before scanning which resolved with reduction of levodopa medication. This person was grouped with the PDnonVH.

Patients were recruited who experienced repetitive and complex visual hallucination symptoms usually reporting seeing these as well‐formed persons, animals or objects, lasting for at least 4 weeks and occurring at least once every 4 weeks. Exclusion criteria were: neurological disorders other than PD; major psychiatric illness including significant symptoms of depression (patients with MADRS >6); presence of cognitive impairment with MMSE < 24/30. All participants were right handed. A levodopa equivalent dose (levodopa and dopaminergic agonists) was calculated with the formula used by Vingerhoets et al. [2002].

Eligible patients were referred by the Movement Disorders Clinic by the attending neurologist, and controls were recruited from the patients' acquaintances and the local community by advertisement. All participants were reimbursed for travel expenses, and signed informed consent for participation. The study was approved by the local ethics Institutional Review Board. Demographic and clinical characteristics are described in Table 1.

**Table 1:** Demographic and clinical profile of HC, PDnonVH, and PDVH

Demographics	HC(*n* = 14)	PDnonVH (*n* = 12)	PDVH (*n* = 12)	*P*‐value
Age	64.1 ± 4.0	63.4 ± 7.4	67.6 ± 7.4	0.234
Gender (females /males)	8/6	8/4	9/3	0.631
Duration of illness (years)	n/a	8.4 ± 5.1	10.0 ± 3.5	0.400
Duration of hallucinations (months)	n/a	n/a	22.6 ± 17.3	n/a
Hoehn and Yahr stage	n/a	2.8 ± 0.9	3.2 ± 0.7	0.160
UPDRS‐III score	n/a	18.0 ± 12.9	20.9 ± 10.6	0.553
Levodopa dose (mg)	n/a	704.9 ± 519.4	978.7 ± 361.3	0.148
Affected body side (R/B/L)	n/a	6/1/5	4/3/5	0.497
MMSE score	29.1 ± 0.7	28.5 ± 1.7	27.6 ± 2.4	0.092

Continuous data are presented in mean ± SD.

MMSE, Mini‐mental State Examination; MADRS‐S, Montgomery–Åsberg Depression Rating Scale—Self‐assessment; UPDRS, Unified Parkinson's Disease Rating Scale; PPRS, Parkinson Psychosis Rating Scale; PANSS, The positive and negative syndrome scale; HC, healthy control; PDnonVH, Parkinson's disease without visual hallucination; PDVH, Parkinson's Disease with visual hallucination. R, right side; L, left side; B, both. n/a = not applicable; *P* values of two group comparisons were calculated using Independent‐Samples *t*‐tests (chi‐squared test for gender and affected body side); *P* values of three group comparisons were calculated using One‐way ANOVA.

**Table 2:** Group difference in functional connectivity of DMN

Direction of difference	Number of voxels	*x*	*y*	*z*	Side	Brain regions	BA	*T*‐value
HC versus PDnonVH								
Lower in PDnonVH	49	27	−30	−12	R	Parahippocampal gyrus/ fusiform gyrus	36/37/20	−3.8709
	58	6	69	3	L/R	Medial prefrontal gyrus	10	−3.4197
	68	−9	−54	3	L	Posterior cingulate gyrus	30/29	−3.4903
	105	−6	−30	27	L	Posterior cingulate gyrus /precuneus	23/31	−3.6918
	85	15	−78	42	R	Precuneus	7	−3.5349
	117	39	−54	33	R	Angular gyrus/ precuneus	39/40	−3.862
	54	27	33	42	R	Middle frontal gyrus	8/9	−3.7874
HC versus PDVH								
Lower in PDVH	46	0	60	6	L/R	Medial prefrontal lobe	10	−2.7296
	56	9	−78	45	R	Precuneus	7	−4.2606
PDnonVH versus PDVH								
Greater in PDVH	164	6	−36	48	L/R	Precuneus/ Posterior cingulate gyrus	31	3.8732
	59	21	42	45	R	Superior middle frontal lobe	8	3.9702

Group functional connectivity differences within DMN are shown at *P* < 0.05 of multiple comparison correction (cluster size = 2619 mm^3^, *t* > 2.07 (or *t* < −2.06)). *x*, *y*, *z*: coordinates in the MNI atlas extending from *z* = −60 mm to +85 mm. BA: Brodmann area. *T* values from a *t*‐test of the peak voxel (showing greatest statistical difference within a cluster), a negative *T* value implies lower functional connectivity within DMN in PDVH. L, R: left and right. In comparisons between HC and both PD groups, age and MMSE score were controlled; in comparison between PDnonVH and PDVH, age, MMSE score and levodopa‐equivalent dosage were controlled.

### MRI Data Acquisition

Resting functional magnetic resonance imaging (MRI) data were acquired by a MRI scanner operating at 3T (Achieva; Philips Healthcare, Hong Kong) using an eight‐channel phased‐array head coil with patients in the supine position. The functional imaging data were acquired using a gradient‐echo echo‐planar imaging sequence sensitive to BOLD contrast [Kwong et al., 1992; Ogawa et al., 1992]. Acquisition parameters were: repetition time (TR) = 1800 ms; echo time (TE) = 30 ms; flip angle (FA) = 90°; 220 volumes; 45 contiguous axial slices; anterior–posterior acquisition; in‐plane resolution = 3.75 × 3.75 mm; slice thickness = 4 mm; field of view (FOV) = 240 × 180 × 240 mm; total acquisition time = 6.6 min. Slice acquisition order was contiguous. Earplugs were used to reduce scanner noise and head motion was restricted by a foam pillow as well as extendable padded head clamps. Before each resting state scan, participants were asked to simply rest in the scanner with their eyes closed and not fall asleep while remaining as still as possible.

Three‐dimensional (3D) T1‐weighted anatomical MRI were also acquired with fast field echo sequence (magnetization prepared rapid gradient echo), with the parameters: TR = 6,895 ms; TE = 3.16 ms; FA = 8°; 1 × 1 × 1 mm voxels; FOV = 250 × 250× 155 mm; number of slices = 155.

### Cortical Thickness Analysis

T1‐weighted structural data was processed using following default procedures of FreeSurfer (version 4.3.1, available at: http://surfer.nmr.harvard.edu) [Dale et al., 1999; Fischl and Dale, 2000; Fischl et al., 1999]. Freesurfer software carries out sulcal alignment and cortical thickness analyses in the spherical space following linear alignment to the Talairach atlas [Talairach and Tournoux, 1988]. This is a two‐dimensional cortical surface‐based coordinate system which has been reported to yield more accurate registration of cortical functional and anatomical areas across individuals than can be attained using the more common 3D coordinate system.[Fischl et al., 1999]. The preprocessing pipeline included: motion correction; linear alignment; bias field effect correction; topological surface defects removal; skull stripping; segmentation into gray matter (GM) and white matter (WM); hemispheres separation; intensity normalization; and subvoxel representation of the GM/WM boundary and pial surfaces. The surface of the GM/WM boundary was inflated and differences between subjects in the depth of gyri and sulci were normalized by nonlinear stretching. Each subject's reconstructed brain was then transformed and registered to an average spherical surface. Maps were smoothed with Gaussian kernel full‐width half maximum (FWHM) of 15 mm.

Cortical thickness was measured based on the shortest distance between GM/WM border and GM/cerebrospinal fluid (CSF) border in each vertex of the cortex. Vertices were organized in triangular grids and assessments of cortical area were obtained by calculating the area of each triangle in a standardized, spherical atlas space surface tessellation when mapped into the individual subject space. Regionally specific between‐group differences in cortical thickness were investigated within the QDEC (query, design, estimate, contrast) application of FreeSurfer with two‐sample *t*‐test models. Monte Carlo simulations were performed on cluster level at *P* < 0.05 (two‐tailed) to correct for multiple comparisons.

### Structural Image Analysis for Functional Analysis

Individual structural T1‐weighted images were coregistered to the mean motion‐corrected functional images using a linear transformation. They were subsequently segmented into GM, WM, and CSF in Montreal Neurological Institute (MNI) space by using “New Segment” in SPM8. DARTEL [Ashburner, 2007] was then used to create a study‐specific template. We opted to use DARTEL in SPM8 instead of FSL for this component of the preprocessing, taking advantage of the high resolution anatomical T1 image of each subject to create a study specific template that is less biased to the control group. DARTEL is a high‐dimensional image registration technique, which allows optimal mapping between subjects [Ashburner, 2007]. It registers all subjects into a common space, where the degree of applied deformation is the same for each individual. After the fMRI images were coregistered with the T1 the rest of the preprocessing procedure followed the standard FSL pipeline. GM, WM, and CSF were normalized to MNI space and smoothed with an 8‐mm FWHM Gaussian kernel. Mean modulated and smoothed GM maps (GM intensity threshold = 0.2) were used to generate a group GM mask and applied as a mask for analyzing functional connectivity differences in the between‐group comparisons, specific to the groups involved in a particular test.

### Functional MRI Data Preprocessing

Preprocessing was performed using SPM8 (http://www.fil.ion.ucl.ac.uk/spm). First, all functional images were corrected for slice timing differences during acquisition then realigned to the first image to correct for head movement. Participants with excessive head movement were discarded, as defined by head motion > 2.5 mm of displacement or > 2.5° of rotation in any direction. Functional data were then normalized to the MNI space by applying the transformation parameters obtained from the structural images (see the above “structural image analysis” section for details) to those time and motion corrected and nuisance covaried images, resampled (3 × 3 × 3 voxels) and smoothed (4‐mm FWHM Gaussian kernel).

### Dual Regression ICA

Multivariate exploratory linear decomposition into independent components (MELODIC) Version 3.09, part of the FSL was used to define probabilistic ICA. Four‐dimensional (4D) preprocessed fMRI data of individuals was concatenated to identify large‐scale patterns of functional connectivity in the study‐specific population. Data were then decomposed into 40 independent components of time and associated 3D spatial maps using automatic dimensionality estimation. The DMN was selected by matching to previous DMN reports [Raichle et al., 2001].

The dual regression approach implemented in FSL (http://fsl.fmrib.ox.ac.uk/fsl/fslwiki/DualRegression) [Beckmann et al., 2005] was applied to identify subject‐specific time courses and spatial maps. This approach is increasingly used to compare large‐scale brain networks between groups [Damoiseaux et al., 2012; Uddin et al., 2013; Veer et al., 2010; Zuo et al., 2010]. The group level spatial ICA maps generated by MELODIC were used for regression to give a set of time‐courses in each individual's 4D data, and the time course matrices were normalized by their variance and used in a linear model fit against each individual fMRI dataset to get individual‐specific spatial maps which reflect the functional coherence within each specific network. The spatial map of DMN was next statistically compared between the three groups (PDnonVH vs. PDVH, PDnonVH vs. HC, and PDVH vs. HC). Between‐group voxel‐wise comparisons were performed within binary mask of the predefined DMN network using two sample *t*‐test with SPM8. The statistical tests were corrected for multiple comparisons to a significance level of *P* < 0.05 using Monte Carlo simulations (uncorrected single voxel significance level of *P* < 0.05 and a minimum cluster size based on the size of the GM mask of each load). [Ledberg et al., 1998].

### Demographic Statistical Analysis

Demographic statistical analysis was performed using Statistical Package for Social Sciences (version 15.0.1; SPSS for Windows, 2006). Independent two sample *t*‐tests were used to compare demographic factors between groups, and chi‐squared tests were used to compare gender and affected body side. In addition, correlation analyses were conducted to reveal any association between the mean functional connectivity *z* score within the DMN in the clusters with significant difference between the two PD groups and visual hallucination severity in PDVH group. Age, MMSE scores, and levodopa‐equivalent dosage were regressed out.

## RESULTS

### Demographic and Clinical Profile

Data from 38 individuals were entered into the analysis. Of these, 24 were patients with PD idiopathic type (comprising 12 with VH and 12 without) and 14 were unaffected healthy controls. The characteristics of the study participants are shown in Table 1.

Clinical data are summarized in Table 1. The PD groups were also balanced for duration of illness, drug dosage and PD symptom severity. In PDVH group, five people reported having VH about 1–3 times per month, five had VH 1–2 times per week, and two reported having VH every day. Nine patients experienced hallucinations of people, one experienced animal and objects, one saw only objects, and one experienced the “presence of a person.” Two patients reported visual illusions in addition to hallucinations. (See Table 1.) Three patients with VH were taking antipsychotic medication (one on mirtazapine, one on quetiapine and one patient on both mirtazapine and quetiapine). Participants were asked if they experienced hallucinations in the scanner—none were reported.

### Cortical Thickness

There were no significant group differences in cortical thickness among three groups.

### Higher Functional Connectivity in the DMN in PDVH Group Compared to PDnonVH Group

**Figure 1:**
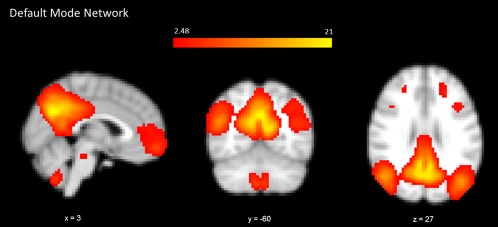
Spatial map representing the DMN for all 38 individuals. Regions belonging to this network include medial prefrontal, posterior cingulate, lateral parietal, and inferior/middle temporal gyri; cerebellar areas; and extending to brainstem regions. [Color figure can be viewed in the online issue, which is available at http://wileyonlinelibrary.com.]

**Figure 2:**
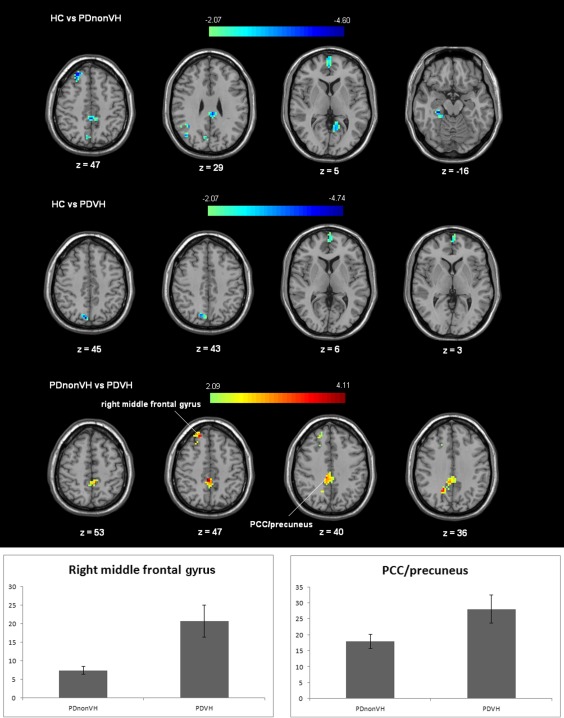
Functional connectivity of DMN differences between groups. The difference maps at the given threshold were corrected for multiple comparisons (Monte‐Carlo Simulation, cluster size = 1215 mm^3^ (45 voxels), *t* > 2.09 (or *t* < −2.09) or *t* > 2.07 (or *t* < −2.07)). In the bottom panel, red/yellow indicates that PDVH participants had greater functional connectivity compared to PDnonVH within the DMN, age, MMSE score and levodopa‐equivalent dosage were controlled in comparison; in the top and middle panel, the blue/green indicates that PDVH or PDnonVH participants had lower functional connectivity than HC within the DMN. The right side of the figure is the left side of the brain. The column bars exhibit the average functional connectivity *z* scores (±standard error) of the two clusters (right middle frontal gyrus, posterior cingulate gyrus /precuneus) with significant difference between PDVH and PDnonVH groups in the two groups. [Color figure can be viewed in the online issue, which is available at http://wileyonlinelibrary.com.]

Probabilistic ICA [Beckmann et al., 2005] defined 40 components representing group‐averaged networks of brain regions with fMRI signals that were temporally correlated. The DMN was identified as that incorporating fMRI signal in the medial prefrontal, posterior cingulate, lateral parietal, and inferior/middle temporal gyri; cerebellar areas; and extending to brainstem regions (Fig. 1) [Di and Biswal, 2014; Raichle et al., 2001]. A significantly higher degree of functional coactivation within the DMN in PDVH patients relative to PDnonVH patients was found in right middle frontal gyrus and bilateral posterior cingulate gyrus/precuneus (Table 2 and Fig. 2).

### Lower Functional Connectivity in the DMN in Both PD Groups Compared to HC

Significantly lower functional connectivity within the DMN in PDnonVH patients compared to HC group was found in right parahippocampal gyrus/fusiform gyrus, bilateral medial prefrontal gyrus, bilateral posterior cingulate gyrus/precuneus, right middle frontal gyrus. Compared to HCs, PDVH patients also showed lower functional connectivity within the DMN in bilateral medial prefrontal lobe and right precuneus.

### Correlation Analysis

In PDVH group, there were no significant correlation between visual hallucination symptom severity and the mean functional connectivity within the two ROIs in DMN.

## DISCUSSION

VH in PD impact upon quality of life [Diederich et al., 2009], and predict cognitive deterioration and mortality [Aarsland et al., 2007; Fenelon et al., 2000]. However, the nature and extent of brain pathology underlying PDVH remains poorly understood. Our main finding was PD reduces functional connectivity in the DMN compared to healthy controls, while patients with VH actually had significantly greater connectivity within the DMN relative to patients without VH.

In PD, the DMN showed reduced connectivity during the resting state, compared to controls. However, connectivity within the PDVH group was greater than the PDnonVH group, particularly across bilateral precuneus/posterior cingulate gyrus and right middle frontal lobe. The decreased functional connectivity within DMN in PD compared to controls is in line with a recent study showing that cognitively unimpaired patients have decreased functional connectivity within the DMN compared to controls [Tessitore et al., 2012]. The authors attributed the DMN deficit to cognitive decline in PD patients because cognitive function and DMN connectivity were correlated. However, in our study, functional connectivity within the DMN was disrupted in PD even after controlling for cognitive function. This suggests that disruption of DMN may be a direct consequence of PD rather than secondary to cognitive deterioration, although we acknowledge that the MMSE is a rather insensitive instrument in this context.

In contrast to the decreased connectivity within the DMN in PD compared to controls, we observed relatively increased connectivity within the DMN in PDVH in both posterior cingulate gyrus/precuneus and frontal regions compared to PDnonVH. This result is consistent with the hypothesis that excessive, or aberrant mnemonic and self‐referential processing contributes to VH in the absence of extrinsic visual stimuli [Olypher et al., 2006]. It is also reminiscent of findings reported in schizophrenia where DMN connectivity differences are proposed to reflect an inability to redirect resources from internal thoughts and feelings toward external stimuli [Whitfield‐Gabrieli et al., 2009]. Since age, cognitive function and levodopa‐equivalent dosage was controlled for, the relatively increased connectivity within the DMN in PDVH is more likely associated with VH than other confounds.

A role for the DMN has been suggested in cognitive decline [Leech and Sharp, 2014; Wang et al., 2013] and depression [Li et al., 2013], however, few studies have investigated its role in psychosis and hallucinations. A recent study found that nonpsychotic individuals with auditory‐verbal hallucinations exhibited greater activity than controls in hub regions such as the temporal cortices and posterior cingulate/precuneus [Lutterveld et al., 2014]. Our results are also broadly similar to the direction of structural connectivity differences reported in individuals with schizophrenia [Amad et al., 2014; Cheung et al., 2011]. The latter studies observed abnormal structural connections in psychosis, but found that individuals with greatest hallucination symptoms actually had “higher” connectivity within the disrupted network, which in turn may increase functional connectivity across distant brain regions [McGrath et al., 2013].

Our connectivity approach provides a useful counterpoint to previous studies of task‐dependent activation patterns reported in previous functional MRI studies in PDVH. Some authors have found hemodynamic differences in DMN regions in PDVH [Ramirez‐Ruiz et al., 2008; Stebbins et al., 2004], while others reported no differences [Holroyd and Wooten, 2006; Ibarretxe‐Bilbao et al., 2011; Meppelink et al., 2009]. Our study, focused on connectivity related to resting activity, avoids some of the potential confounds arising from differences in task design and/or difficulty. This illustrates the advantage of employing a resting‐state design in clinical disorders where cognitive decline may be an important confound, but a corollary is that task‐dependent and resting‐state studies are not directly comparable.

### Limitations

We acknowledge that our sample size made type II error a possibility. For example, we conducted an exploratory correlation analysis of relationship between DMN connectivity in PDVH and severity of hallucinations and although the relationship was positive, this did not reach statistical significance. The negative findings from our cortical thickness analysis may also result from type II error and thus subtle structural differences which might accompany functional anomalies in the PDVH group cannot be discounted. That said, this is a difficult population to recruit for MRI scanning and although our number compares favorably to the existing literature, a MRI follow‐up study with greater statistical power may help decipher negative findings.

We recognize that an initial three‐group ANCOVA would have been an alternative approach to the analysis, followed by post hoc t‐contrasts. However, our pairwise approach better reflects our nested hypotheses. Our primary focus was on key differences in the DMN of individuals with PD who experience hallucinations, and those who do not. We considered that hallucinations in PD are not simply due to more severe illness and a direct comparison of PDnonVH and PDVH groups would be the most optimally controlled experiment. We then included a HC to better understand general differences between PD groups and HCs (lower FC in DMN in PD with and without hallucinations) and to show that those with PD and hallucinations were not those simply with the “worst” DMN abnormality, but formed a distinct subgroup. The greater connectivity within the DMN of those with PD and hallucinations was in the opposite direction to more general lower DMN connectivity in PD, suggesting the functional anatomy of hallucinations in PD is quite specific to those individuals with these particular symptoms.

We caution against over‐generalization of our findings. The individuals in the PDVH group were grossly cognitively intact and in a relatively early stage of the illness. Their VH ranged between “mild” and “moderate” in severity. Therefore, we cannot say whether our results will extend to those affected by more severe symptoms.

Finally, the PD groups in our study were scanned “on” their usual dopaminergic medication. The “off” state can cause discomfort, episodic high amplitude movement leading to decreased signal‐to noise ratio. Although dopaminergic medication may contribute to VH in PD, the levodopa dosage was controlled in our study design. Therefore any effects of levodopa medication effect were unlikely to be the principal explanations of the group effects we observed.

## CONCLUSIONS

Our study demonstrates for the first time that VH in PD are associated with higher functional connectivity within the DMN, relative to patients without hallucination, and that this occurs in the absence of significant cortical atrophy. Our study opens up the possibility that examination of the DMN in PD could offer some predictive utility for those who may be at risk of developing VH and consequently an accompanying poor prognosis. This enhances the efforts to move toward targeting more stratified treatment approaches for individuals with PD [Park and Stacy, 2011].
